# Photoreceptor Specificity in the Light-Induced and COP1-Mediated Rapid Degradation of the Repressor of Photomorphogenesis SPA2 in Arabidopsis

**DOI:** 10.1371/journal.pgen.1005516

**Published:** 2015-09-14

**Authors:** Song Chen, Niels Lory, Johannes Stauber, Ute Hoecker

**Affiliations:** Botanical Institute and Cluster of Excellence on Plant Sciences (CEPLAS), Biocenter, University of Cologne, Cologne, Germany; University of Geneva, SWITZERLAND

## Abstract

The Arabidopsis COP1/SPA E3 ubiquitin ligase is a key negative regulator that represses light signaling in darkness by targeting transcription factors involved in the light response for degradation. The COP1/SPA complex consists of COP1 and members of the four-member SPA protein family (SPA1-SPA4). Genetic analysis indicated that COP1/SPA2 function is particularly strongly repressed by light when compared to complexes carrying the other three SPAs, thereby promoting a light response after exposure of plants to extremely low light. Here, we show that the SPA2 protein is degraded within 5–15 min after exposure of dark-grown seedlings to a pulse of light. Phytochrome photoreceptors are required for the rapid degradation of SPA2 in red, far-red and also in blue light, whereas cryptochromes are not involved in the rapid, blue light-induced reduction in SPA2 protein levels. These results uncover a photoreceptor-specific mechanism of light-induced inhibition of COP1/SPA2 function. Phytochrome A (phyA) is required for the severe blue light responsiveness of *spa* triple mutants expressing only SPA2, thus confirming the important role of phyA in downregulating SPA2 function in blue light. In blue light, SPA2 forms a complex with cryptochrome 1 (cry1), but not with cryptochrome 2 (cry2) *in vivo*, indicating that the lack of a rapid blue light response of the SPA2 protein is only in part caused by a failure to interact with cryptochromes. Since SPA1 interacts with both cry1 and cry2, these results provide first molecular evidence that the light-regulation of different SPA proteins diverged during evolution. SPA2 degradation in the light requires COP1 and the COP1-interacting coiled-coil domain of SPA2, supporting that SPA2 is ubiquitinated by COP1. We propose that light perceived by phytochromes causes a switch in the ubiquitination activity of COP1/SPA2 from ubiquitinating downstream substrates to ubiquitinating SPA2, which subsequently causes a repression of COP1/SPA2 function.

## Author Summary

Plants have evolved photoreceptors that initiate a signaling cascade to adjust growth and development to the ambient light environment. The CUL4-dependent COP1/SPA E3 ubiquitin ligase is a key negative regulator of light signaling whose function is repressed by light. Recent research has identified mechanisms that are common to both phytochrome and cryptochrome photoreceptors. Here, we have identified a mechanism of light-induced COP1/SPA repression that is specific to phytochrome photoreceptors. We show that the SPA2 protein is very rapidly degraded in red, far-red and blue light in a phytochrome-dependent fashion. We further show that SPA2 degradation in the light depends on COP1 and on the interaction of SPA2 with COP1. Hence, our results suggest a light-induced degradation of SPA2, but not of COP1, by the COP1/SPA2 ubiquitin ligase. The human ortholog of COP1, which functions without the plant-specific SPA proteins, is known to be regulated by autodegradation following DNA damage. Hence, autodegradation of components of this E3 ligase is a regulatory mechanism used in both humans and plants.

## Introduction

As sessile organisms plants continuously monitor the ambient light conditions and adjust their growth and development with the aim to optimize growth and—ultimately—seed production in a competitive environment. Plants sense the intensity, color, direction and periodicity of light. Responses to these light parameters include seedling deetiolation (inhibition of hypocotyl elongation, opening of cotyledons and apical hook, greening), phototropism, shade avoidance, the accumulation of anthocyanins and the induction of flowering in particular day lengths [1].

To sense the light, plants have evolved several classes of photoreceptors [1,2]. The phytochrome photoreceptors sense red light (R) and far-red light (FR) and exist in two R/FR photointerconvertible conformations. Among the five phytochromes in Arabidopsis (phyA-phyE), the relatively light-stable phyB is the primary phytochrome controlling FR-reversible responses to R. These responses are also named low fluence responses (LFR). phyA is rapidly degraded in R and primarily mediates high-irradiance responses (HIR) to continuous FR (FRc) and very low fluence responses (VLFR) [3,4]. Blue light (B) is sensed by cryptochromes, phototropins and the ZEITLUPE family, but also by phyA. Cryptochromes are encoded by two genes in Arabidopsis, *CRY1* and *CRY2*. Both mediate seedling deetiolation in B, while primarily cry2 is responsible for B-induced flowering in long days [5,6]. For both, phytochromes and cryptochromes, mutant photoreceptor variants have been identified that are constitutively active and thus signal also in darkness [7–10]. Recently, UVR8 was identified as the long-sought UV-B receptor [11,12].

In Arabidopsis, the phytochrome and cryptochrome photoreceptors act to inhibit a key repressor of light signaling that prevents light responses in darkness. This repressor, the CONSTITUTIVELY PHOTOMORPHOGENIC1/SUPPRESSOR OF PHYA-105 (COP1/SPA) complex, functions as an E3 ubiquitin ligase which ubiquitinates positively-acting light signaling intermediates, mainly transcription factors, thereby targeting them for proteolytic degradation in the 26S proteasome. In the light, photoreceptors directly interact with the COP1/SPA complex, leading to its inactivation which subsequently allows the target transcription factors to accumulate and to initiate vast reprogramming of gene expression [13,14]. The degradation of the light-labile photoreceptors phyA and cry2 is also in part dependent on *COP1* and/or *SPA* genes [15–18]. The Arabidopsis COP1/SPA complex is likely a tetramer consisting of two COP1 and two SPA subunits [19]. *COP1* is a single-copy gene in higher plants, while SPA proteins are encoded by a small gene family of four genes in Arabidopsis (*SPA1-SPA4*) and 2 genes in rice [13,20]. Mutations in either *COP1* or all four *SPA* genes lead to constitutive photomorphogenesis in Arabidopsis, with seedlings showing the features of light-grown seedlings in complete darkness [21,22]. While *cop1* null mutants arrest growth at the seedling stage, *spa* null mutants are viable. *cop1 spa* quintuple null mutants can complete embryogenesis, indicating that the COP1/SPA complex is not necessary for embryogenesis [23]. Apart from controlling seedling growth, the COP1/SPA complex also plays an important role during other light-induced responses, such as anthocyanin biosynthesis, elongation responses during shade avoidance, leaf expansion and the suppression of flowering under non-inductive short-day conditions. These responses are mediated through a number of COP1/SPA substrates including CO, HFR1, PAP1, PAP2 and BBX family proteins [24–32]. Moreover, COP1/SPA is a positive regulator in UV-B mediated photomorphogenesis [11,12]. The four *SPA* genes have overlapping but also distinct functions in controlling the various light responses during plant development [22,24–26,33].

The COP1/SPA complex acts as part of a CULLIN4 (CUL4)-based E3 ubiquitin ligase. CUL4-associated E3 ligases consist of CUL4, RBX1, DDB1 as well as a variable WD repeat protein which recognizes the substrate and binds DDB1 [34,35]. The WD repeat proteins COP1 and SPA are substrate adaptors in CUL4-DDB1^COP1/SPA^ E3 ligase(s) [36]. Both COP1 and SPAs contain a central coiled-coil domain responsible for the formation of the COP1/SPA complex via homo- and heterodimerization [19,37,38]. In their C-termini, both COP1 and SPAs carry a WD-repeat domain which mediates interaction with substrates as well as with DDB1 [36,39]. The N-termini of COP1 and SPA are distinct, with COP1 harboring a RING finger domain and SPA proteins carrying a kinase-like domain [40,41].

Light is the key factor controlling COP1/SPA activity. Genetic studies showed that the SPA2 protein is particularly strongly inactivated by light when compared to the other three SPAs, making SPA2 a particularly interesting SPA when analyzing light-mediated inhibition of COP1/SPA activity [22,42]. How light inactivates the COP1/SPA complex is not fully understood. Evidence indicates that phytochrome and cryptochrome photoreceptors converge on COP1/SPA to promote light signaling in R, FR and B. Such light-induced inactivation of COP1/SPA occurs via multiple mechanisms. First, after light exposure, COP1 translocates from the nucleus into the cytoplasm [43,44]. Second, the B-dependent interaction of cry1 with SPA1 reduces the COP1/SPA1 interaction [45–47]. Similarly, an interaction of light-activated phytochromes A and B with members of the SPA family reduces the interaction between COP1 and SPA proteins [48,49]. For cry2, B acts to promote the interaction of cry2 with COP1 [50]. A third mechanism which reduces COP1/SPA activity in FRc-grown plants involves the degradation of SPA1 and SPA2 in the proteasome [42]. Here, we have analyzed the molecular mechanism of SPA2-degradation in different light qualities and uncover a photoreceptor-specific mechanism of light-induced COP1/SPA repression via COP1.

## Results

### SPA1 and SPA2 are degraded in far-red, red and blue light

**Fig 1:**
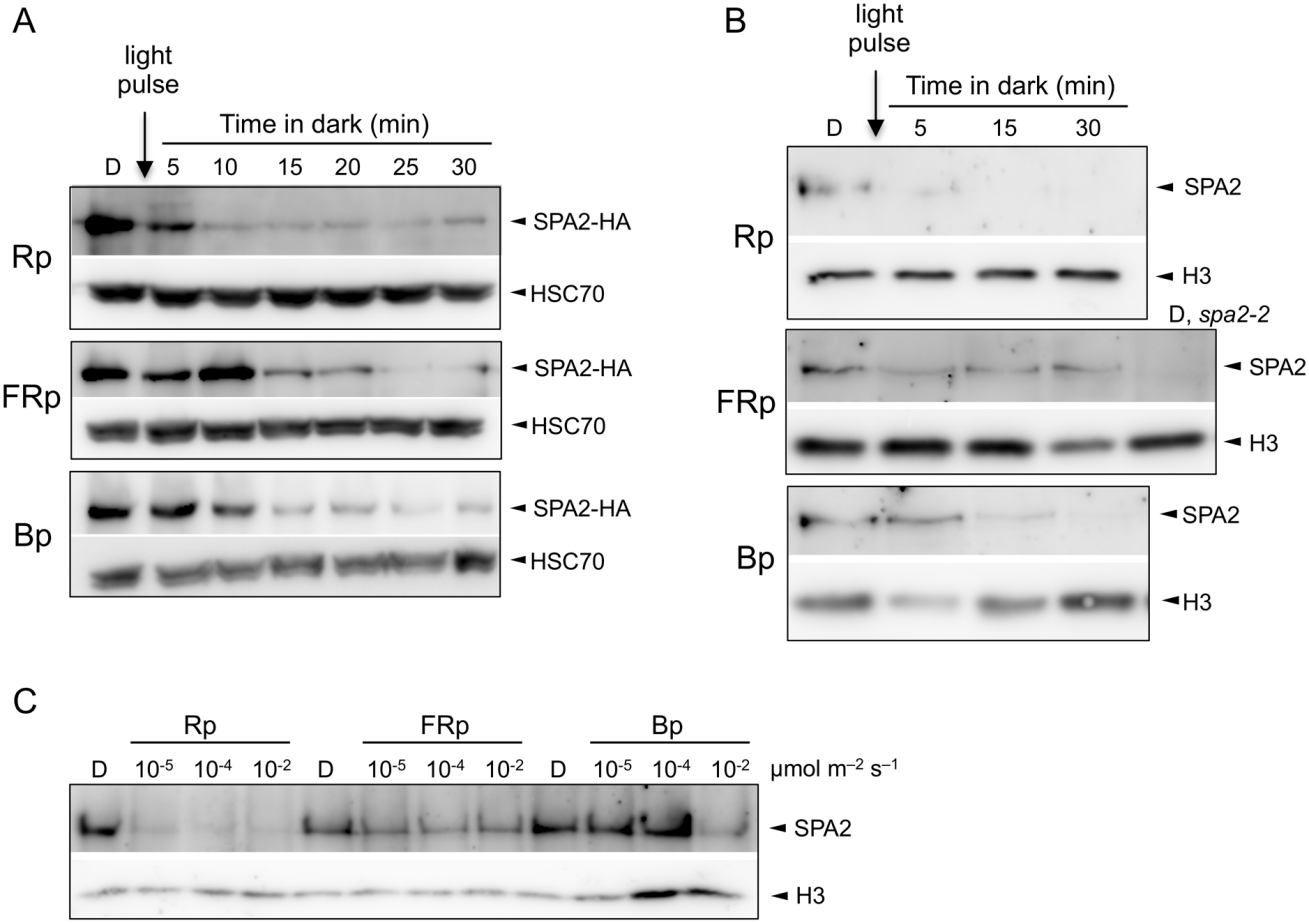
Light exposure rapidly reduces SPA2 protein levels. **A.** SPA2-HA protein levels in 4-day-old dark-grown (D) seedlings that were irradiated with a 200-s-pulse of 0.01 μmol m^–2^ s^–1^ FR (FRp), 0.01 μmol m^–2^ s^–1^ R (Rp) or 0.1 μmol m^–2^ s^–1^ B (Bp) and subsequently kept in the dark for the indicated time. Seedlings carried the *SPA2*::*SPA2-HA* transgene in a *spa1 spa2 spa3* background. Proteins were detected using α-HA and α-HSC70 antibodies (loading control). **B.** SPA2 protein levels in 4-day-old dark-grown (D) wild-type seedlings that were irradiated with a 200-s-pulse of 0.01 μmol m^–2^ s^–1^ FR (FRp), 0.01 μmol m^–2^ s^–1^ R (Rp) or 0.2 μmol m^–2^ s^–1^ B (Bp) and subsequently kept in the dark for the indicated time. Proteins were detected in nuclear extracts using α-SPA2 antibodies. Histone H3 levels (H3) served as a loading control. **C.** SPA2 protein levels in wild-type seedlings that were transferred to a 200-s-pulse of R, FR or B of the indicated fluence rates and subsequently kept in darkness for 2 h. Proteins were detected in nuclear extracts using α-SPA2 antibodies. Histone H3 levels (H3) served as a loading control.

To investigate the dynamics and wave-length dependency of light-induced SPA2 degradation, we determined SPA2 protein levels in dark-grown seedlings that were briefly exposed to R, FR or B. These seedlings expressed HA-tagged SPA2 under the control of the 5′ and 3′ regulatory sequences of *SPA2* (*SPA2*::*SPA2-HA*) [42]. The *SPA2* promoter expresses at the same level in dark-grown and light-exposed seedlings [42,51]. Therefore, light-induced differences in SPA2-HA protein levels in these lines are due to changes in protein stability, as shown previously [42]. Exposure of dark-grown seedlings to a short, 200-second pulse of R (Rp) was sufficient to strongly reduce SPA2-HA protein levels within 5 min after subsequent transfer to darkness (Fig 1A). Ten minutes after the Rp, there was barely any SPA2-HA protein detectable. Similarly, when dark-grown seedlings were irradiated with a pulse of FR (FRp) or B (Bp), SPA2-HA protein abundance decreased to a very low level. The response time to FRp and Bp was also very rapid, but slightly longer when compared to Rp.

To determine whether the native SPA2 protein behaves like the SPA2-HA protein, we analyzed SPA2 protein levels in wild-type seedlings using an α-SPA2 antibody. Because SPA2 levels are very low, we enriched the protein preparations through nuclear extracts to detect the constitutively nuclear-localized SPA2 protein [22,42]. Fig 1B shows that a pulse of FR, R or B rapidly and strongly reduced SPA2 protein abundance. Again, Rp was more effective in reducing SPA2 levels than FRp and Bp. We subsequently asked what fluences are necessary for the reduction of SPA2 protein levels. Fluences of 0.002 μmol m^-2^ of R, i.e. a 200-s-pulse of R with a fluence rate of 10^−5^ μmol m^-2^ s^-1^, was sufficient to reduce SPA2 protein levels to almost undetectable levels (Fig 1C), indicating that degradation in R is extremely sensitive to light and likely involves a VLFR. FRp and Bp were again less effective than Rp (Fig 1C).

**Fig 2:**
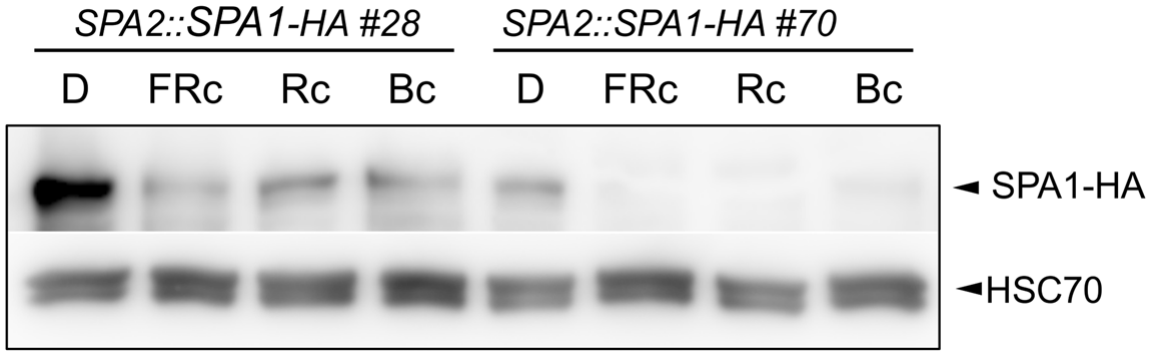
Light exposure reduces SPA1 protein levels. SPA1-HA protein levels in transgenic seedlings expressing SPA1-HA under the control of the native *SPA2* promoter. Seedlings were grown in darkness (D) for 4 days and subsequently transferred to FRc (5 μmol m^–2^ s^–1^), Rc (10 μmol m^–2^ s^–1^) or Bc (50 μmol m^–2^ s^–1^) for 6 h. SPA1-HA was detected using an α–HA antibody. HSC70 levels served as a loading control.

We subsequently asked whether R, FR and B also cause a decrease in SPA1 levels. Here, the induction of *SPA1* gene expression by light [40] precluded a specific analysis of protein stability using α-SPA1 antibodies. Therefore, we used transgenic lines expressing SPA1-HA under the control of the constitutive *SPA2* promoter. These lines showed a strong reduction in SPA1-HA abundance in FR, R and B (Fig 2).

### Rapid SPA2 degradation in R, FR and B is exclusively mediated by phytochromes

**Fig 3:**
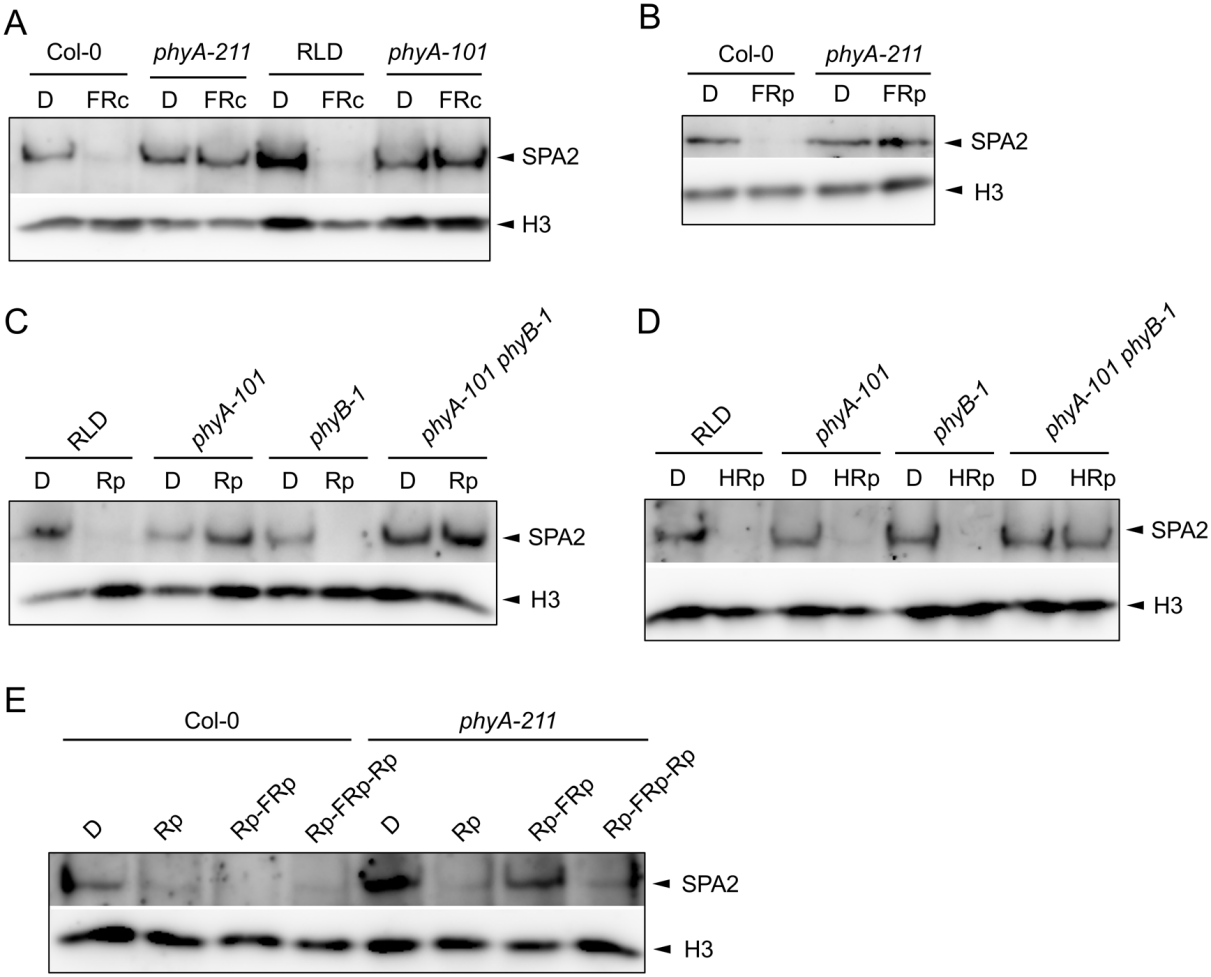
SPA2 degradation in FR and R requires phytochromes and is R/FR reversible. **A.** SPA2 protein levels in 4-day-old dark-grown wild-type and *phyA* mutant seedlings that were transferred to FRc (0.1 μmol m^–2^ s^–1^) for 30 min. *phyA-211* is in Col and *phyA-101* in RLD accession. **B.** SPA2 protein levels in 4-day-old dark-grown wild-type Col-0 and *phyA-211* mutant seedlings that were irradiated with a 200-s-pulse of 5 μmol m^–2^ s^–1^ FR (FRp) and subsequently kept in the dark for 30 min. **C, D.** SPA2 protein levels in the 4-day-old dark-grown seedlings of wild-type RLD, *phyA-101*, *phyB-1* and *phyA-101 phyB-1* mutants that were irradiated with a 200-s-pulse of 0.01 μmol m^–2^ s^–1^ R (Rp)**(C)** or 10 μmol m^–2^ s^–1^ R (HRp)**(D)** and subsequently kept in the dark for 30 min. **E.** SPA2 protein levels in wild-type Col-0 and *phyA-211* mutant seedlings that were grown in darkness for 4 days and subsequently irradiated with a 200-s-pulse of 10 μmol m^–2^ s^–1^ R (Rp), a 200-s-pulse of 10 μmol m^–2^ s^–1^ R followed by a 200-s-pulse of 5 μmol m^–2^ s^–1^ FR (Rp-FRp) or a 200-s-pulse of 10 μmol m^–2^ s^–1^ R in addition to Rp-FRp (Rp-FRp-Rp). After irradiation, seedlings were kept in the dark for 30 min. SPA2 levels were detected in nuclear extracts using an α-SPA2 antibody. Histone H3 levels (H3) served as a loading control.

We asked which photoreceptor(s) are responsible for degradation of SPA2 in different light qualities and quantities. To this end we investigated SPA2 levels in various photoreceptor mutants. Degradation of SPA2 in response to FRc was fully abolished in a *phyA* mutant, in both Col and RLD accessions (Fig 3A). Similarly, a pulse of FR had no effect on SPA2 protein levels in a *phyA* mutant (Fig 3B). Hence, phyA is responsible for SPA2 degradation in FR.

After a pulse of R with low fluence rates, SPA2 protein levels were not reduced in a *phyA* mutant nor in a *phyA phyB* double mutant. A *phyB* mutant, in contrast, showed a reduction in SPA2 levels after Rp and thus exhibited a similar response as the wild type (Fig 3C). The phyA-requirement for a response to low Rp confirms that this treatment initiates a phyA-perceived VLFR [3]. After a pulse with higher fluence rates of R, only the *phyA phyB* double mutant lacked a reduction in SPA2 protein abundance when compared to dark-grown seedlings (Fig 3D). Hence, phyA and phyB mediate the degradation of SPA2 after high Rp. This suggests that both VLFR and LFR responses trigger SPA2 degradation in red light. R/FR reversibility is a hall-mark of an LFR [4]. Indeed, SPA2 degradation after Rp was reversible by a pulse of FR in a *phyA* mutant background which would lack the VLFR (Fig 3E).

**Fig 4:**
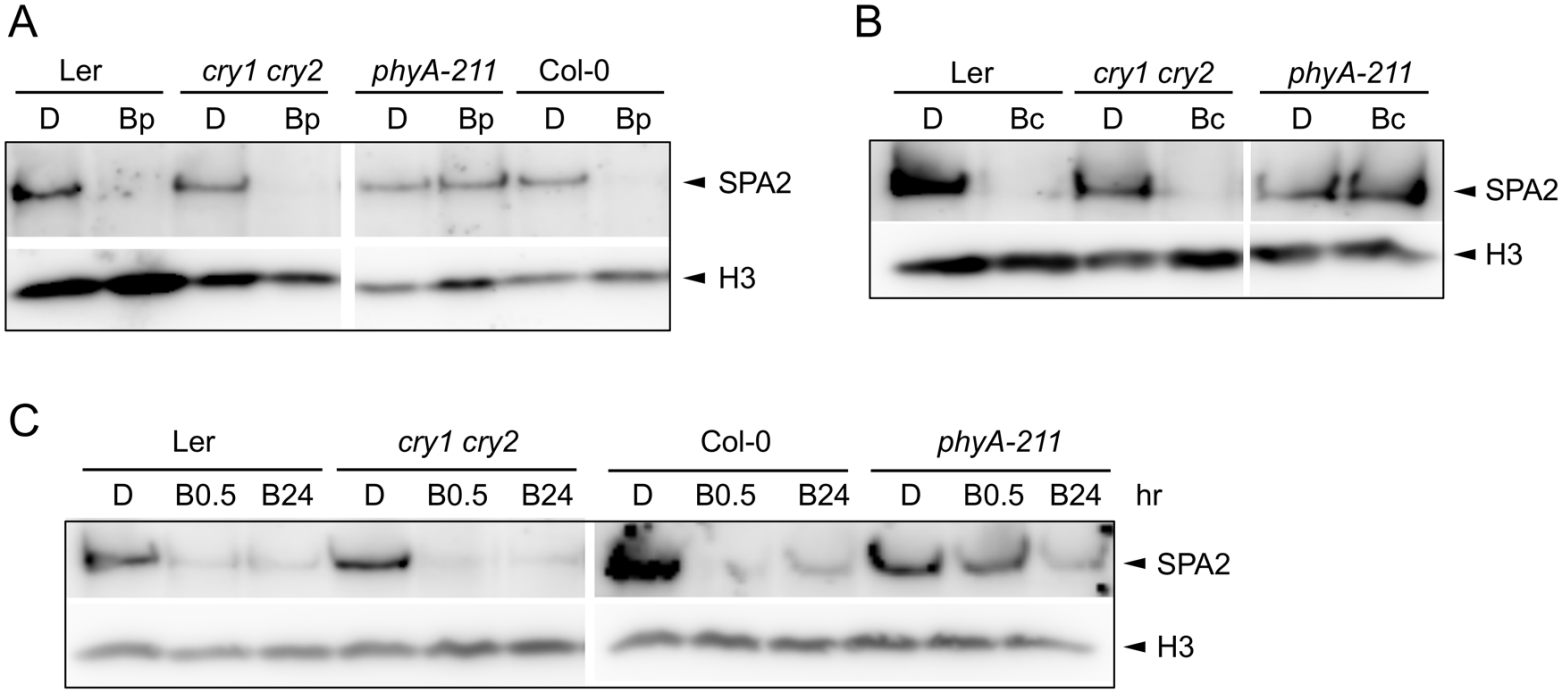
Degradation of SPA2 in B primarily requires phyA. **A-C.** SPA2 protein levels in 4-day-old dark-grown (D) seedlings of the indicated genotypes that were **(A)** irradiated with a 200-s-pulse of 0.2 μmol m^–2^ s^–1^ B (Bp) and subsequently kept in the dark for 30 min, **(B)** transferred to Bc (50 μmol m^–2^ s^–1^) for 30 min or **(C)** transferred to 20 μmol m^–2^ s^–1^ Bc for 0.5 h (B0.5) or 24 h (B24). SPA2 levels were detected in nuclear extracts using an α-SPA2 antibody. Histone H3 levels (H3) were used as a loading control.

Deetiolation in blue light is mediated by the cryptochromes cry1 and cry2 as well as by phyA. We therefore investigated B-induced degradation of SPA2 in *cry1 cry2* and in *phyA* mutants. After a pulse of B, the decrease in SPA2 levels was abolished in *phyA* mutant seedlings but was normal in the *cry1 cry2* mutant (Fig 4A). These results indicate that a pulse of B only triggered phyA-mediated SPA2 degradation. Also after irradiation with continuous B of very high fluence rates (50 μmol m^-2^ s^-1^) for 30 min high SPA2 levels were retained in *phyA* mutant seedlings. In *cry1 cry2* mutant seedlings, SPA2 levels were again strongly reduced similar to wild-type seedlings (Fig 4B). Only after prolonged irradiation with B of high fluence rates for 24 h, SPA2 levels decreased in a phyA-deficient mutant (Fig 4C). These results show that the rapid B-induced reduction in SPA2 levels is exclusively mediated by phyA. Only after very long irradiation with B of high fluence rates other photoreceptor(s) become active in reducing SPA2 levels.

In an attempt to uncover a possible role of cryptochromes in the response to long-term B irradiation, we analyzed SPA2 protein levels in a *cry1 cry2 phyA-201* triple mutant background (Ler accession). SPA2 protein levels still decreased in this mutant after prolonged exposure to blue light (S1A Fig). However, SPA2 levels in *phyA-201* also decreased after FRc (S1B Fig). Hence, degradation of SPA2 in the *cry1 cry2 phyA-201* triple mutant may either be due to residual phyA activity or, alternatively, the regulation of SPA2 stability may be different in the Ler accession than in the Col and RLD accessions.

### SPA2 levels in transgenic lines expressing constitutively active photoreceptors

**Fig 5:**
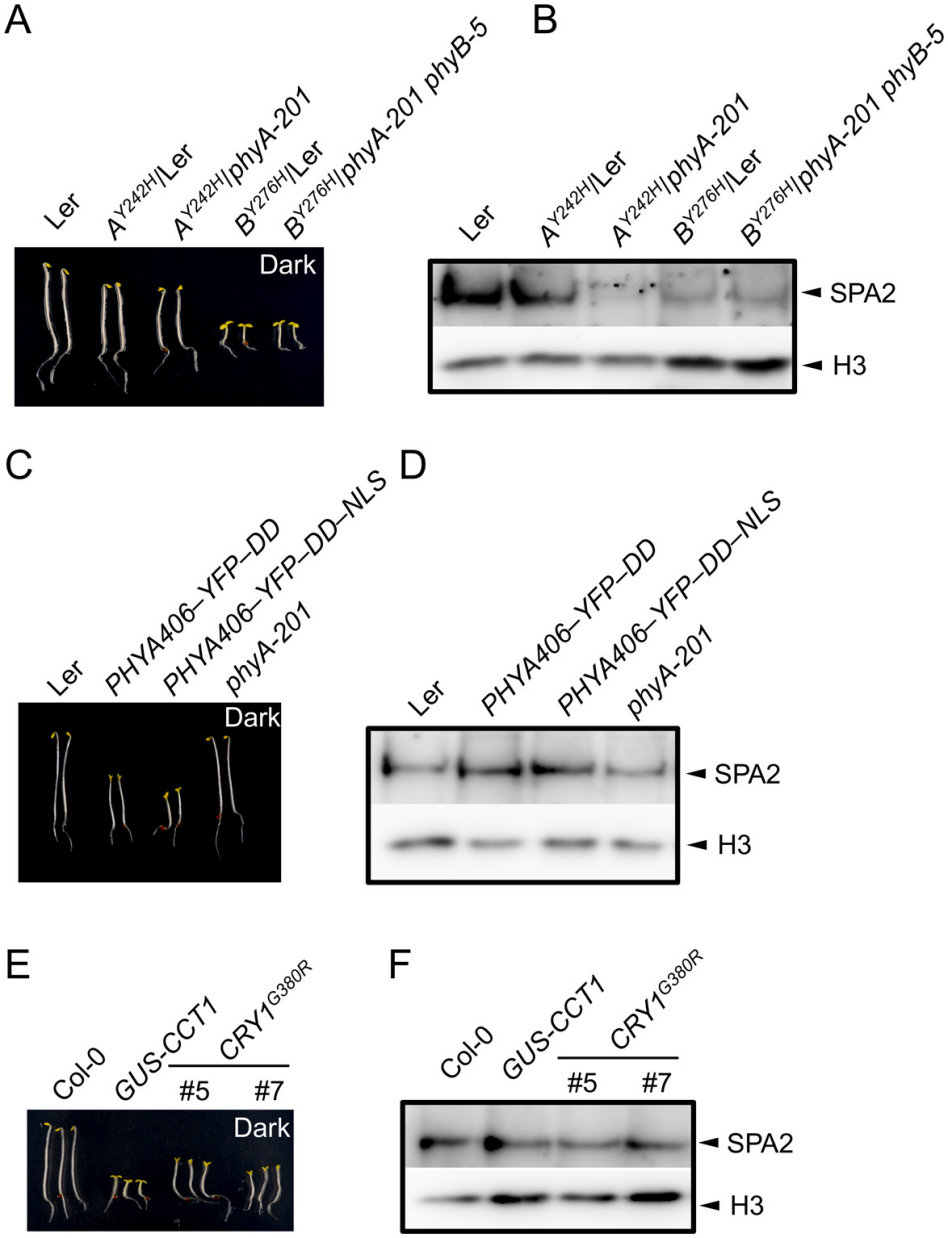
SPA2 protein levels in transgenic seedlings expressing constitutively active photoreceptors. **A, C, E.** Phenotype of 4-day-old dark-grown seedlings of the indicated genotypes. Both *PHYA406* constructs were in the *phyA-201* background. **B, D, F.** Immunodetection of SPA2 protein levels in 4-day-old dark-grown seedlings. SPA2 was detected in nuclear extracts using α-SPA2 antibodies. Histone H3 levels (H3) served as a loading control.

Constitutively active photoreceptor variants have been described that initiate light signaling even in darkness. We therefore investigated whether these photoreceptor variants also cause a constitutive reduction in SPA2 protein abundance, i.e. also in dark-grown seedlings. To this end, we analyzed SPA2 protein levels in transgenic lines expressing the constitutively active phytochrome mutants phyB^Y276H^ and phyA^Y242H^ [7]. As reported previously, phyB^Y276H^-expressing seedlings showed very strong constitutive photomorphogenesis, both in the *PHYB* wild-type and the *phyB-5* mutant background [7] (Fig 5A). These phyB^Y276H^ lines showed very low SPA2 protein levels in dark-grown seedlings (Fig 5B), suggesting that the SPA2 protein is destabilized in darkness by the constitutively active phyB photoreceptor.

phyA^Y242H^-expressing seedlings exhibit weaker constitutive photomorphogenesis than phyB^Y276H^-expressing seedlings [7]. These seedlings had a shorter hypocotyl and a partially opened hook, especially in the *phyA* mutant background, when compared to the wild type (Fig 5A). This phenotype was somewhat weaker than reported previously which is likely due to the younger age of our seedlings and the absence of sucrose in the culture medium when compared to [7]. SPA2 abundance in dark-grown seedlings was strongly reduced in lines expressing phyA^Y242H^ in a *phyA* background, while it was similar to wild type in lines expressing phyA^Y242H^ in a *PHYA* wild-type background (phyA^Y242H^/Ler) (Fig 5B). Hence, SPA2 levels were constitutively reduced in the presence of phyA^Y242H^, but this effect is outcompeted by the presence of wild-type phyA. In summary, these mutations in both phyA and phyB cause constitutive degradation of SPA2 in darkness.

Expression of the N-terminal 406 amino acids of phyA fused to an artificial dimerization domain has also been shown to cause constitutive photomorphogenesis in darkness [10] (Fig 5C). However, this phyA variant did not alter SPA2 protein levels in darkness (Fig 5D), indicating that this constitutively active phyA variant was not capable of inducing SPA2 degradation in darkness.

Fusion of the cry1 C-terminal extension (CCT1) to an artificial dimerization domain (GUS) leads to a constitutively active cry1 photoreceptor. Similarly, a cry1^G380R^ variant is constitutively active. Hence, seedlings expressing CCT1 or cry1^G380R^ exhibit strong constitutive photomorphogenesis in darkness [8,9]. SPA2 protein levels were unaltered in dark-grown GUS-CCT1- and cry1^G380R^-expressing seedlings when compared to the wild type, despite the constitutive photomorphogenesis displayed by these seedlings (Fig 5E and 5F). Hence, none of the constitutively active cry1 variants affected SPA2 protein levels in darkness. This is in agreement with the primary roles of phytochromes in SPA2 degradation.

### B-induced reduction in SPA2 function in *spa1 spa3 spa4* mutants strongly depends on phyA

**Fig 6:**
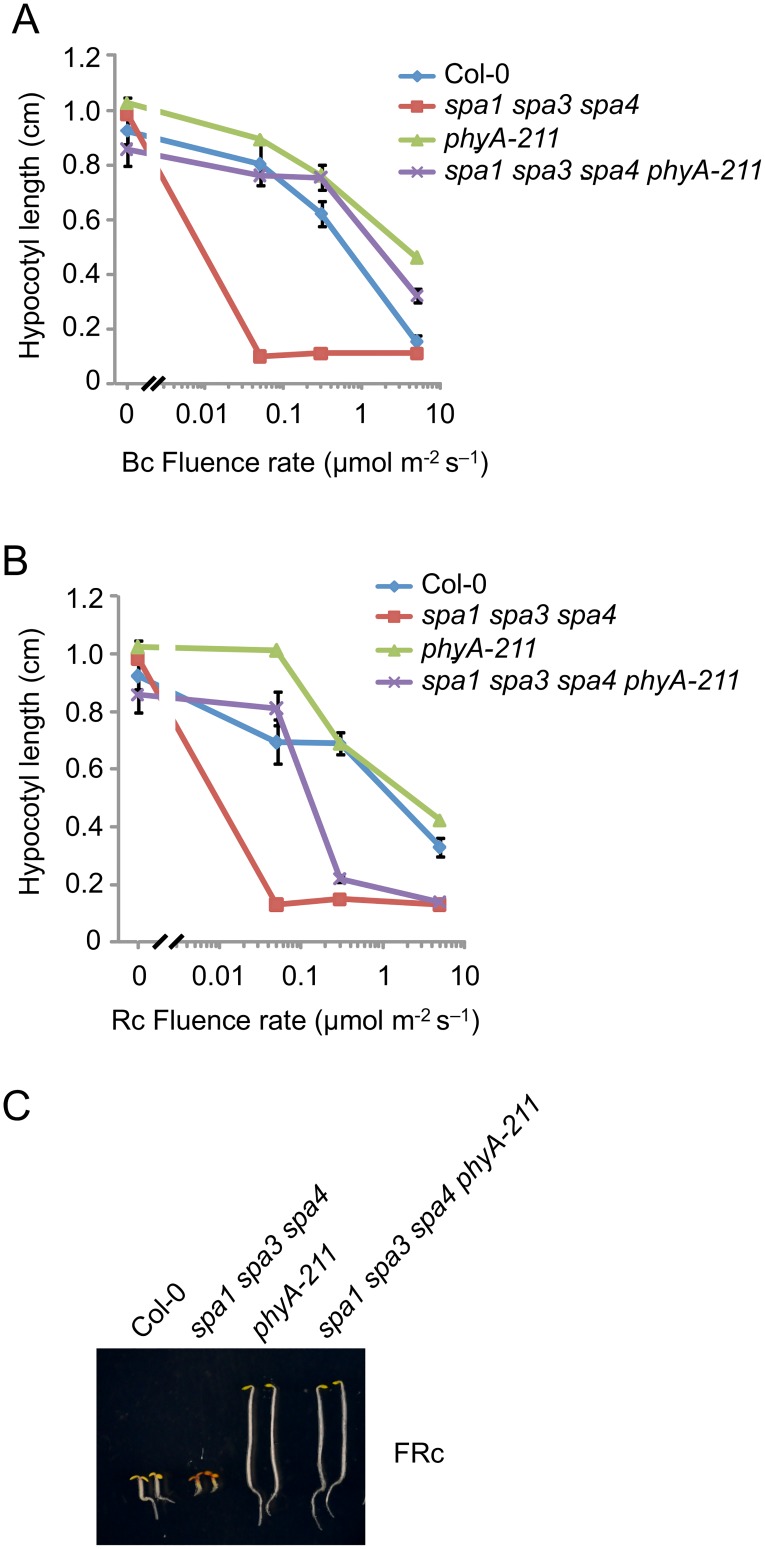
The *phyA-211* mutation abolishes the hypersensitivity of the *spa1 spa3 spa4* mutant to B and FR but not to R. **A, B.** Hypocotyl length of seedlings of the indicated genotypes grown in various fluence rates of Bc (**A**) and Rc (**B**) for 4 days. Error bars represent the SEM. **C.** Visual phenotype of seedlings of the indicated genotypes grown in FRc (2 μmol m^–2^ s^–1^) for 4 days.

Since rapid degradation of SPA2 in B was exclusively dependent on phyA, we predicted that phyA is of particular importance in inactivating SPA2 function in B. To test this hypothesis, we generated a phyA-deficient *spa1 spa3 spa4 phyA* mutant which only expresses functional SPA2 among the four SPA proteins. Hence, we can observe the effect of light on SPA2 activity in the absence of any other SPAs, and in the presence or absence of phyA. We had shown previously that *spa1 spa3 spa4* mutant seedlings etiolate normally in darkness but are very hypersensitive to R, FR and B when compared to the wild type, thus resembling a *spa* quadruple mutant already at extremely low fluence rates of light [22,42] (Fig 6A–6C). Hence, SPA2 is sufficient for full repression of photomorphogenesis in darkness but is extremely effectively inactivated by light. In B, *spa1 spa3 spa4 phyA* mutant seedlings displayed much longer hypocotyls than *spa1 spa3 spa4* mutant seedlings, indicating that the lack of phyA dramatically reduced the responsiveness of the *spa1 spa3 spa4* mutant to B. The hypocotyl length of the *spa1 spa3 spa4 phyA* mutant in B was very similar to that of the *phyA* single mutant. Hence, in the absence of phyA, the mutations in *SPA1*, *SPA3* and *SPA4* had no detectable effect (Fig 6A). In Rc, the *phyA* mutation abolished the hypersensitivity of the *spa1 spa3 spa4* mutant to lower fluence rates of Rc but not to higher fluence rates of Rc (Fig 6B). This is consistent with our finding that SPA2 degradation in lower fluence rates of R requires phyA, while in higher fluence rates of R phyB in addition to phyA mediates SPA2 degradation. As expected, the responsiveness of *spa1 spa3 spa4* mutant seedlings to FRc was fully dependent on phyA (Fig 6C). Taken together, these results show that the hypersensitivity of the *spa1 spa3 spa4* mutant to B fully depends on phyA. This agrees with our observation that rapid SPA2 degradation in B was exclusively dependent on phyA. Since the *spa1 spa3 spa4 phyA* mutant retained responsiveness to B, as indicated by the inhibition of hypocotyl elongation in B of higher fluence rates, additional phyA-independent mechanisms of SPA2 inactivation by B exist. These are likely mediated by the cryptochromes.

### SPA2 associates with cry1 but not with cry2 in B-treated seedlings

**Fig 7:**
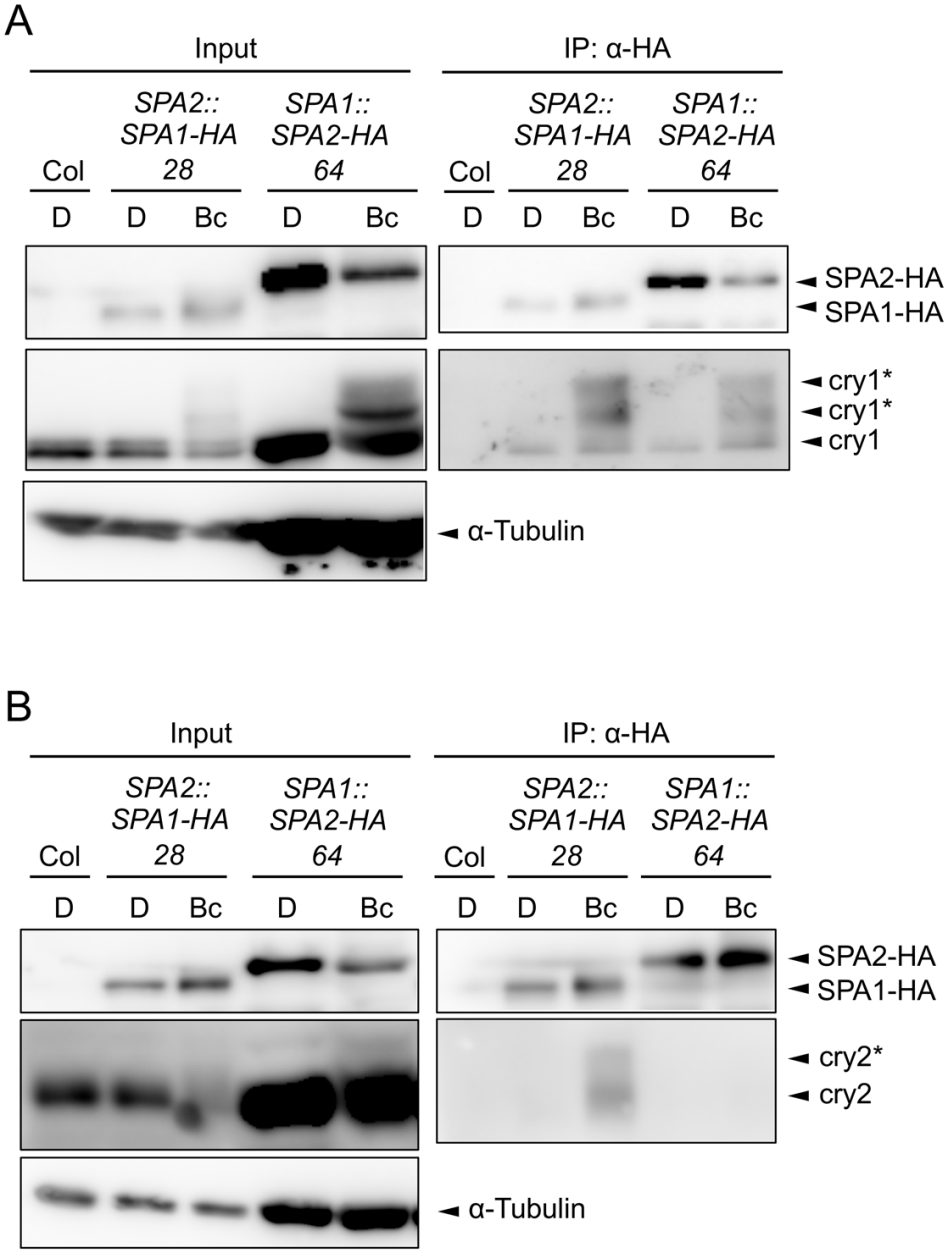
SPA2 associates with cry1 in B but not with cry2. **A, B.** Co-immunoprecipitation of cry1 (**A**) and cry2 (**B**) by SPA1-HA and SPA2-HA. 4-day-old dark-grown seedlings (D) expressing SPA1-HA or SPA2-HA were transferred to 50 μmol m^–2^ s^–1^ Bc for 1 h (**A**) or 5 min (**B**). SPA1-HA and SPA2-HA proteins were immunoprecipitated using α-HA beads. To obtain similar protein levels of SPA1-HA and SPA2-HA in Bc, SPA1-HA was expressed under the control of the weaker *SPA2* promotor (*SPA2*::*SPA1-HA*) and SPA2-HA from the stronger *SPA1* promoter (*SPA1*::*SPA2-HA*). Moreover, seedlings were treated with proteasome inhibitor to prevent SPA degradation in Bc, and five times more protein extract was used for the SPA2-HA immunoprecipitation than for the SPA1-HA immunoprecipitation. α-HA antibody was used to detect SPA-HA proteins. α-cry1 and α-cry2 antibodies were used to detected cry1 and cry2, respectively. α-tubulin levels were used as loading control for the input. Asterisks likely indicate phosphorylated cry1 and cry2.

The lack of cryptochrome activity in B-induced SPA2 degradation might be caused by a failure of cryptochromes to rapidly interact with SPA2. Indeed, FRET/FLIM studies in transfected tobacco leaves failed to show an interaction between cry2 and SPA2. Similarly, recombinantly produced cry2 and SPA2 did not interact in *in vitro* pull-down assays [15]. On the contrary, SPA2 was shown to weakly interact with cry2 in B in the yeast two-hybrid system [50]. For cry1, no significant interaction with SPA2 was observed in the yeast two-hybrid assay [46]. To reinvestigate this question *in planta*, we conducted co-immunoprecipitation experiments using transgenic Arabidopsis seedlings expressing SPA2-HA and, as a positive control, SPA1-HA (Fig 7). To obtain similar protein levels of SPA1-HA and SPA2-HA in B, SPA1-HA was expressed under the control of the weaker *SPA2* promoter (*SPA2*::*SPA1-HA*) and SPA2-HA from the stronger *SPA1* promoter (*SPA1*::*SPA2-HA*). Moreover, seedlings were treated with proteasome inhibitor to reduce SPA degradation in B. Fig 7A shows that upon B-exposure both SPA1-HA and SPA2-HA co-immunoprecipitated higher-mobility cry1 isoforms which are formed in B. Hence, B induced the formation of a SPA2/cry1 complex, as it was previously reported for a SPA1/cry1 complex [46,47]. In addition, a lower-mobility cry1 which likely represents the non-phosphorylated isoform of cry1 showed weak constitutive interactions with SPA1-HA and SPA2-HA in B and darkness. The association of higher-mobility cry1 with SPA2 was very rapid. It occurred within 5 min of B-exposure (S2 Fig). cry2, in contrast, was not co-immunoprecipitated by SPA2-HA, neither in darkness nor in B. The positive control SPA1-HA showed the expected B-dependent association with cry2 (Fig 7B). In summary, in B, SPA2 associates with cry1 but not with cry2 *in planta*.

### COP1 is necessary for the light-induced degradation of SPA2

**Fig 8:**
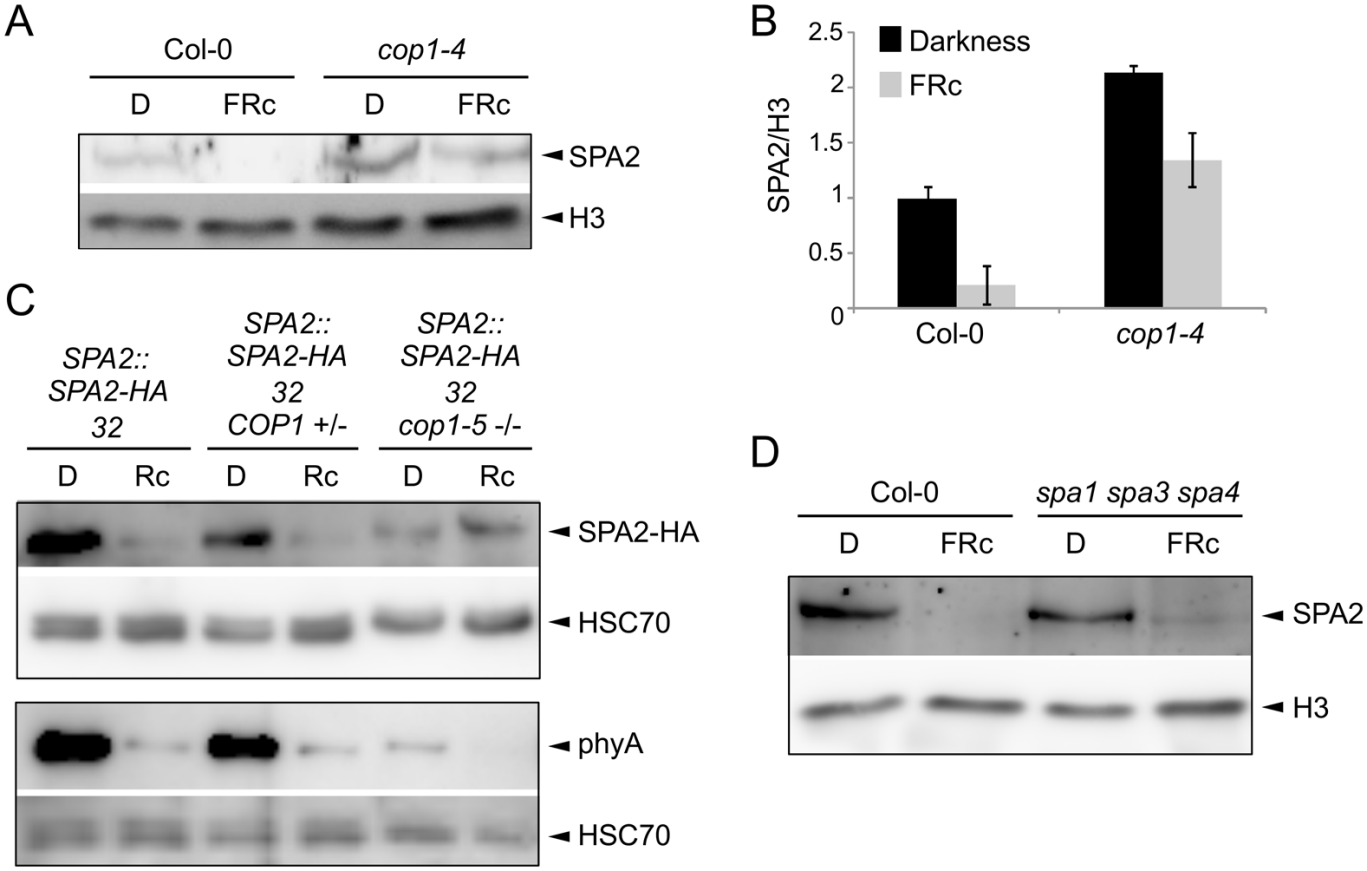
COP1 is required for SPA2 degradation in the light. **A, B.** Immunodetection (**A**) and quantification (**B**) of SPA2 protein levels in 4-day-old wild-type Col-0 and *cop1-4* mutant seedlings grown in darkness (D) or in FRc (0.35 μmol m^–2^ s^–1^). The error bars represent the SEM of two biological replicates. **C.** SPA2-HA protein levels in the *cop1-5* null mutant background. The *SPA2*::*SPA2-HA 32* transgene was crossed into a *cop1-5* mutant background. *SPA2*::*SPA2-HA 32 cop1-5 (-/-)* represents homozygous *cop1-5* mutants selected from a segregating population based on the black seed phenotype. *SPA2*::*SPA2-HA 32 COP1 (+/-)* represents homozygous *COP1* wild-type or heterozygous *COP1 cop1-5* siblings of the same segregating population. Seeds were incubated in darkness for 1 day and subsequently transferred to Rc (40 μmol m^–2^ s^–1^) for 6 h or kept in darkness. **D.** Immunodetection of SPA2 protein levels in 4-day-old wild-type Col-0 and *spa1-100 spa3-1 spa4-3* mutant seedlings grown in darkness (D) or FRc (0.35 μmol m^–2^ s^–1^). Native SPA2 was detected in nuclear extracts using an α-SPA2 antibody. SPA2-HA and phyA were detected using an α-HA and an α-phyA antibody, respectively. Histone H3 levels (H3) or HSC70 levels were used as loading controls.

To identify the E3 ubiquitin ligase that mediates SPA2 degradation in the light, we asked whether the COP1/SPA E3 ligase itself may be responsible for ubiquitination of SPA2. We therefore investigated SPA2 protein levels in the hypomorphic *cop1-4* mutant and in the *cop1-5* null mutant, using light conditions that cause full degradation of SPA2. In a *cop1-4* hypomorphic background, considerable SPA2 protein levels were retained in seedlings irradiated with FRc (Fig 8A and 8B). Hence, the FRc-induced reduction in SPA2 protein abundance was strongly attenuated, but not abolished, by the partial-loss-of-function *cop1-4* mutation. We subsequently analyzed SPA2 protein levels in the *cop1-5* null mutant. Because *cop1* null mutants arrest growth at the very early seedling stage and, moreover, mostly fail to break the seed coat during germination, we could not obtain enough tissue for nuclear-enriched protein preparations which are necessary to detect the native SPA2 protein with α-SPA2 antibodies. We therefore crossed the *SPA2*::*SPA2-HA* transgene into a *cop1-5* mutant background and detected the SPA2-HA protein using α-HA antibodies. This transgene-encoded SPA2-HA fully mimics function and behavior of the native SPA2 protein [42] (this study). As shown in Fig 8C and asreported above, SPA2-HA protein levels in the progenitor *SPA2*::*SPA2-HA* line decreased to almost undetectable levels upon irradiation with Rc. In a homozygous *cop1-5* mutant background, in contrast, SPA2-HA levels were not reduced in Rc when compared to darkness. As an additional control, we also determined SPA2-HA protein levels in *COP1* wild-type siblings that segregated in a progeny derived from the cross of *cop1-5* with the *SPA2*::*SPA2-HA* line. In these siblings, SPA2-HA protein levels decreased upon Rc irradiation as in the progenitor *SPA2*::*SPA2-HA* line. Hence, the Rc-induced reduction in SPA2-HA protein abundance was fully dependent on COP1.

Because the *cop1* null mutations severely affect seedling growth and cause growth arrest, we wished to exclude the possibility that premature lethality is an indirect reason for the lack of SPA2 degradation in *cop1-5*. To do so, we made use of previous findings showing that degradation of phyA-Pfr is in part COP1-independent [16,18] and thus should occur in *cop1-5*. Indeed, phyA levels strongly decreased upon Rc irradiation (Fig 8C). Hence, the *cop1-5* tissue used was clearly still capable of light perception and light response. The analysis of phyA abundance indicated that phyA levels were considerably lower in *cop1-5* than in the wild type in both dark-grown and light-exposed tissues. The reasons for this are unknown. It may relate to the low efficiency of protein extraction using *cop1-5* when compared to using the wild type. Hence, normalization to HSC70 levels may be unreliable. Consistent with this idea, SPA2-HA levels were also unexpectedly lower in *cop1-5* than in the wild type.

Since the four SPA proteins heterodimerize in the tetrameric COP1/SPA complex [19], we asked whether the presence of SPA1, SPA3 and SPA4 affects SPA2 protein levels in the light. The light-induced reduction in SPA2 protein levels was also dramatic in the *spa1 spa3 spa4* mutant, but slightly higher SPA2 protein levels consistently remained in FRc in *spa1 spa3 spa4* when compared to the wild type (Fig 8D). Hence, the COP1/SPA2 complex which forms in the *spa1 spa3 spa4* mutant is sufficient to allow SPA2 degradation in the light. Whether SPA2 is required for its own degradation cannot be determined from the presented experiments. The finding that the other three SPAs slightly increase SPA2 degradation in FRc hints at the possibility that SPA2 is involved in its own degradation.

### The COP1-interacting coiled-coil domain of SPA2 is necessary for SPA2 degradation

**Fig 9:**
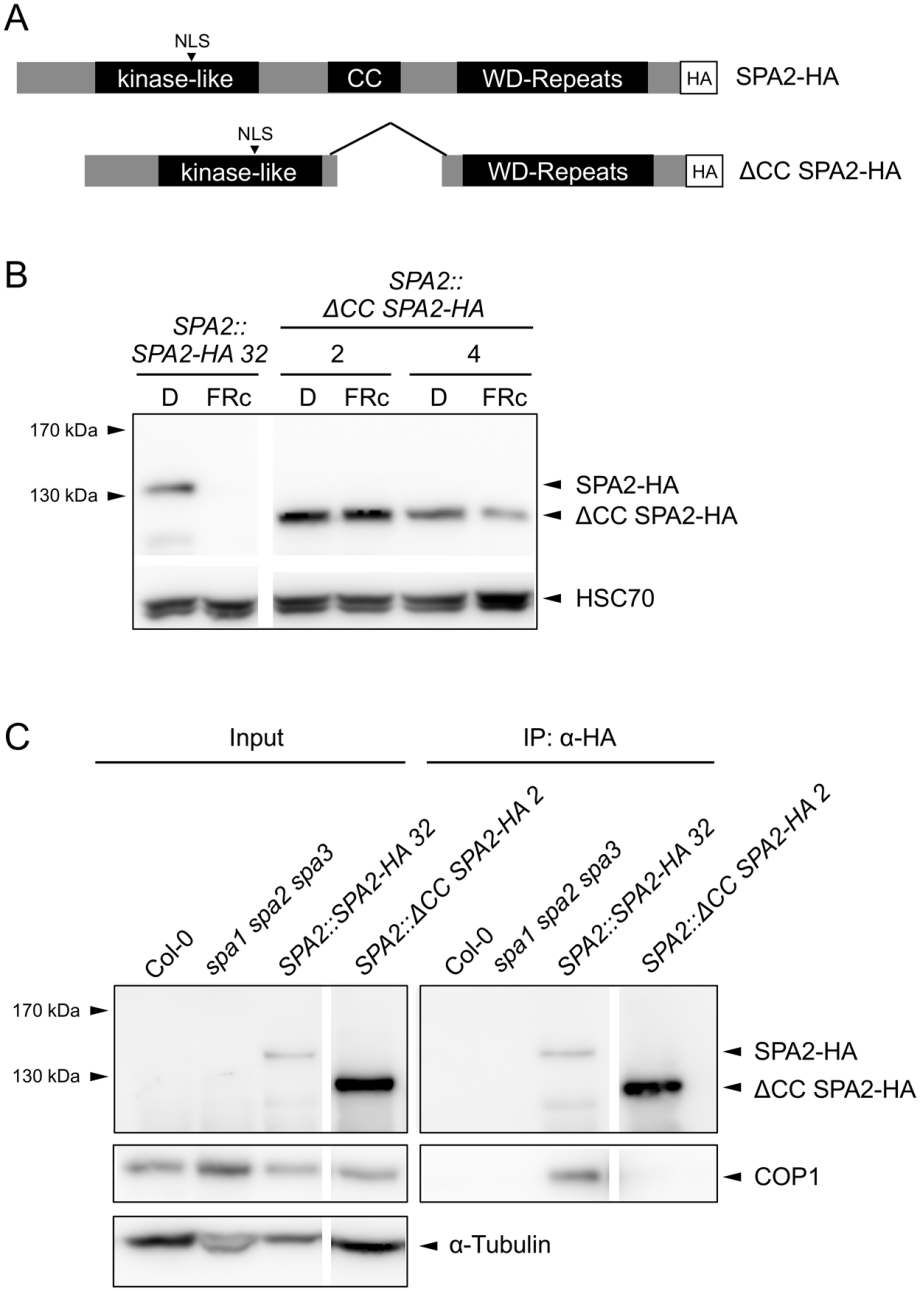
The coiled-coil domain of SPA2 is required for the interaction of SPA2 with COP1 and for SPA2 degradation in the light. **A.** Schematic representation of the full-length SPA2-HA and the ΔCC SPA2-HA proteins. All proteins were expressed in transgenic *spa1 spa2 spa3* mutant plants under the control of the native *SPA2* promoter. **B.** SPA2-HA and ΔCC SPA2-HA protein levels in transgenic *spa1 spa2 spa3* mutant seedlings grown in darkness or FRc (5 μmol m^–2^ s^–1^) for 4 days. SPA-HA proteins were detected using an α–HA antibody. HSC70 levels were used as a loading control. Line numbers represent independent transgenic lines. **C.** ΔCC SPA2-HA does not interact with COP1. SPA2-HA and ΔCC SPA2-HA were immunoprecipitated by α–HA beads. Wild-type Col-0 and the *spa1 spa2 spa3* mutant were used as negative controls. SPA-HA proteins were detected by an α–HA antibody. COP1 proteins were detected by an α-COP1 antibody. α-tubulin levels were used as a loading control for the input fractions. Line numbers represent independent transgenic lines.

Our finding that COP1 is required for SPA2 degradation in the light suggests that SPA2 is directly ubiquitinated by the COP1 or COP1/SPA2 ubiquitin ligase. If so, it is expected that interaction of SPA2 with COP1 is necessary for SPA2 degradation to occur. To test this hypothesis, we expressed a SPA2 deletion derivative that lacks the COP1-interacting coiled-coil domain under the control of the native *SPA2* promoter (*SPA2*::Δ*CC SPA2-HA*; Fig 9A). Indeed, the ΔCC SPA2-HA protein failed to co-immunoprecipitate COP1 in extracts of transgenic plants, confirming that ΔCC SPA2-HA does not incorporate into a COP1/SPA complex (Fig 9C). Consistent with this finding, the Δ*CC SPA2-HA* transgene did not complement the *spa1 spa2 spa3* mutant phenotype, whereas the full-length *SPA2-HA* transgene did (S3 Fig). ΔCC SPA2-HA protein abundance did not change in response to light (Fig 9B). The levels of full-length SPA2-HA, in contrast, decreased to undetectable levels in FRc. This difference in the behavior of the SPA2-HA and ΔCC SPA2-HA proteins is not due to any differences in *SPA2-HA* and Δ*CC SPA2-HA* transcript levels because transcript levels were not regulated by light, as expected for a gene expressed from the *SPA2* promoter (S4 Fig). These results show that the COP1-interacting coiled-coil domain of SPA2 is necessary for SPA2 degradation in the light.

## Discussion

The four SPA proteins are components of the COP1/SPA E3 ubiquitin ligase and have redundant but also distinct functions in regulating plant growth and development in response to the light environment. The phenotypic analysis of *spa* mutants showed that SPA2, among the four SPA proteins, exhibits the greatest difference in activity between dark- and light-grown seedlings and is therefore a particularly interesting SPA protein when investigating light-induced inactivation of COP1/SPA activity [22,42]. Here, we have analyzed the molecular mechanism of SPA2 degradation in different light qualities and have uncovered a photoreceptor-specific mechanism of light-induced COP1/SPA repression via COP1.

Our results demonstrate that the SPA2 protein is degraded very rapidly, i.e. within 5–15 min after dark-grown seedlings were exposed to a brief pulse of R, FR or B. Since COP1 function depends on SPA proteins, this rapid, light-induced degradation of SPA2 provides a very effective mechanism to inactivate COP1/SPA2 activity in light-grown plants. We and others have shown previously that COP1 levels do not significantly change in response to R, FR or B [41,42]. Hence, light does not affect the stability of the whole COP1/SPA2 complex but only that of SPA2. This shows that the presence of SPA2 in the COP1/SPA2 E3 ubiquitin ligase provides a means for light-induced inactivation of the COP1/SPA2 complex. Though both phytochrome and cryptochrome photoreceptors inactivate COP1/SPA function in the respective light qualities [14], we found that the rapid degradation of SPA2 specifically required phytochromes not only in R and FR, but also in B. Thus, this mechanism of rapid COP1/SPA2 inactivation is specific to phytochrome action. In summary, our analysis shows that a photoreceptor-specific mechanism of COP1/SPA2 inactivation developed during evolution. Evidence indicates that multiple mechanisms have evolved that inactivate COP1/SPA function in the light. Another mechanism of inactivation was found to be common to phytochromes and cry1 since phyA, phyB and cry1 induce a dissociation of COP1 from SPA1 in R or B, respectively [46–49]. A third mechanism, the light-induced exclusion of COP1 from the nucleus also occurs in R, FR and B and is primarily mediated by phyA, phyB and cry1 in FR, R and B, respectively [52]. On the other hand, B-control of COP1 nuclear abundance was found to also require biosynthesis of the phytochrome chromophore [53], suggesting an essential role of phytochromes also in B. In total, evidence indicates that photoreceptor-specific mechanisms and common mechanisms induced by both phy and cry photoreceptors co-act to allow an appropriate response to a changing light environment.

Our results show that rapid SPA2 degradation in R involves a phyA-dependent VLFR and a phyB-dependent LFR which is also reversible by FR. In FR, SPA2 degradation was fully dependent on phyA. This demonstrates that the responsiveness of the SPA2 protein to R and FR directly correlates with our current knowledge on phyA and phyB activities in R and FR [3,4] and thus appears to be an immediate output of light-induced phytochrome action. Previous findings showing that SPA2 directly interacts with phyA and phyB [49] are in good agreement with this conclusion. phyA is also a well-known B-photoreceptor that together with cry1 and cry2 is responsible for seedling deetiolation in B [4]. The particular biological significance of phyA in B-induced repression of SPA2 function is supported by our finding that the extreme hypersensitivity to B in *spa1 spa3 spa4* triple mutants which only have functional SPA2 was indeed fully dependent on phyA. We therefore suggest that light inactivates COP1/SPA2 function in B primarily through rapid, phyA-induced degradation of SPA2. Residual SPA2 protein that escapes degradation may be inactivated by additional mechanisms, such as cry1-mediated dissociation from COP1, as it has been described for SPA1 [46,47], and phyA-mediated dissociation from COP1 [49]. The latter, however, has not been analyzed in B so far.

Since the SPA1 protein is also degraded in R, FR and B, albeit with lower efficiency than SPA2, a SPA1-containing COP1 complex may also be inactivated through phytochrome-mediated degradation of SPA1, i.e. via the same or a very similar mechanism as the light-induced degradation of SPA2. Interestingly, the mutant phenotypes of *spa* single mutant seedlings defective in *SPA1*, *SPA3* or *SPA4* are also fully dependent on phyA, even in R. These single mutants etiolate normally in darkness, but exhibit hypersensitivity in the light in a *PHYA* wild-type background only [33,54,55]. The mechanistic reason for this observation has so far remained unknown but could be explained by a phyA-mediated de-stabilization of these SPA proteins in light-grown seedlings. Hence, a stabilization of SPA1, SPA3 and SPA4 in a *phyA* mutant background might lead to the complete rescue of the *spa* single mutant phenotypes.

The failure of other B receptors than phyA, such as cryptochromes, to cause rapid degradation of SPA2 in B is not due to a general lack of SPA2-cry interactions in vivo. However, our results demonstrate that SPA2 only associates with cry1 and not with cry2 in B-treated seedlings. Hence, the lack of a cry2-SPA2 interaction is likely in part responsible for the observed stability of SPA2 in B-treated *phyA* mutant seedlings. On the other hand, our results also show that SPA2 rapidly interacts with cry1 in B without causing rapid SPA2 degradation. Based on this finding we conclude that the failure of cry1 to cause rapid degradation of SPA2 is not due to a lack of a SPA2-cry1 interaction, especially since the SPA2-cry1 interaction is observed rapidly in vivo, i.e. within 5 min of B irradiation. Thus, cry1 interacting with SPA2 in B does not induce rapid degradation of SPA2; cry1 action thereby strongly differs from phytochrome actions on the SPA2 protein.

In contrast to SPA2 which only interacted with cry1 in our *in vivo* co-immunoprecipitation experiments, SPA1 interacted with both cryptochromes, as shown previously [46,47,50]. Hence, SPA1 and SPA2 clearly differ in their interaction capacity with cry2. cry2 was shown to interact with the N-terminal domain of SPA1 [50]. Though we do not know the cry2-interacting domain in SPA2, it is possible that the relatively high sequence divergence between the N-terminal domains of SPA1 and SPA2 might be the cause for their differential interaction capacities with cry2. cry1, in contrast, interacts with the WD-repeat domains of SPA1 and SPA2 [47], and this domain is highly conserved between SPA1 and SPA2 [55].

**Fig 10:**
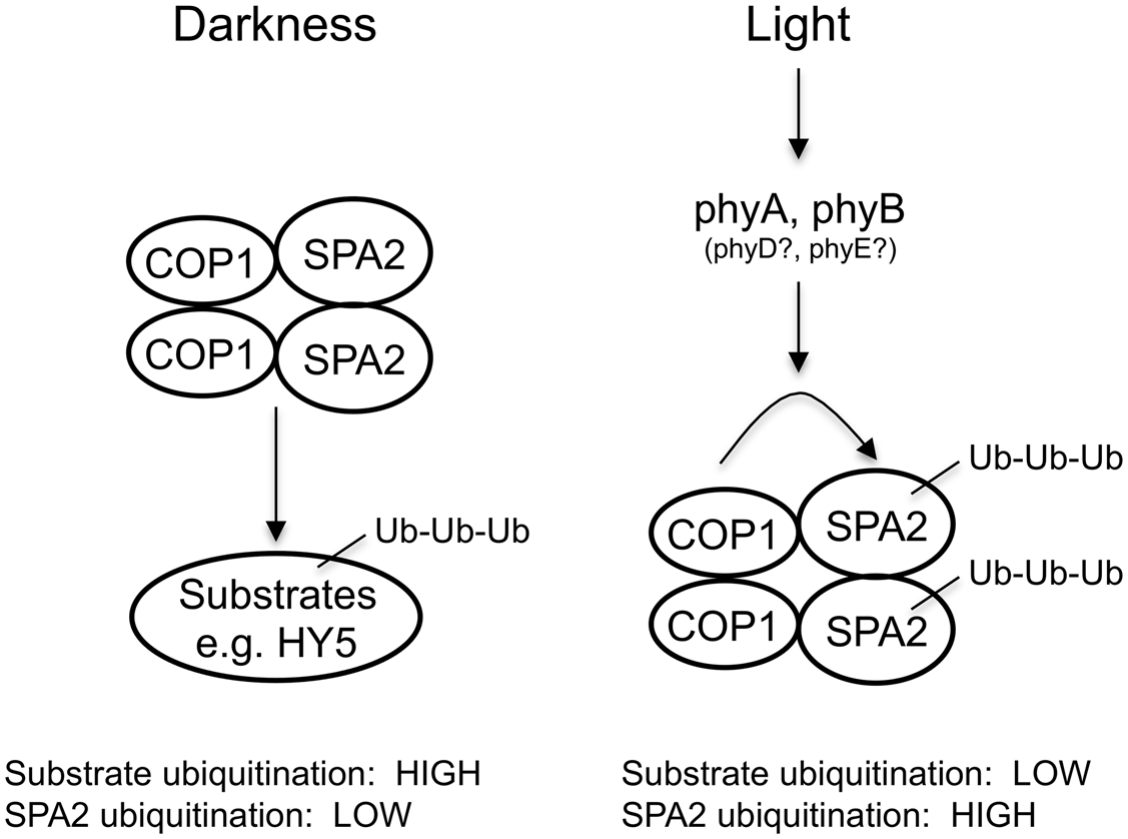
Model for the effect of light on the ubiquitination activities of the COP1/SPA2 E3 ubiquitin ligase.

The mechanism of SPA2 degradation may essentially reflect ubiquitination by the COP1 (or COP1/SPA2) E3 ubiquitin ligase or the action of another E3 ligase. Recently, the COP1-interacting E3 ubiquitin ligase COP1 SUPPRESSOR1 (CSU1) was reported to de-stabilize COP1 and SPA1 in darkness, but not in the light. SPA2, SPA3 and SPA4 protein levels were not altered in *csu1* mutants, neither in dark-grown nor in light-grown seedlings [56]. It is therefore unlikely that CSU1 is involved in the light-dependent degradation of SPA2. Indeed, light-induced SPA2 degradation was absent in a *cop1-5* null mutant. Hence, ubiquitination of SPA2 by COP1 or the COP1/SPA2 ubiquitin ligase is the likely mechanism. This is supported by our finding that a ΔCC SPA2 deletion derivative which does not interact with COP1 *in vivo* is not degraded in the light. We therefore propose that light influences the E3 ligase activity of COP1/SPA2 in two ways: it inhibits COP1/SPA2 E3 ligase activity towards its substrate transcription factors, while it enhances COP1 (or COP1/SPA2) (auto)-ubiquitination activity towards SPA2 and, possibly, SPA1 as well (Fig 10). However, we cannot fully exclude the possibility that SPA2 is ubiquitinated by an indirect COP1-dependent mechanism. For example, COP1 might be a scaffolding protein required for SPA2 degradation or control the activity of another E3 ubiquitin ligase. Whether SPA3 and SPA4 protein stability is controlled by light remains to be determined. In humans, DNA damage increases COP1 autodegradation by ATM-mediated phosphorylation of COP1, followed by stabilization of the COP1 substrate p53 as a cell cycle check point [57]. Though the phosphorylated residue in human COP1 is not conserved neither in Arabidopsis COP1 nor in the SPA proteins, this finding shows that autodegradation of components of this E3 ligase is a regulatory mechanism used in both humans and plants.

## Materials and Methods

### Plant materials, light sources and growth conditions

Wild-type *Arabidopsis thaliana* accessions Col-0, RLD and Ler were used in this study. Photoreceptor mutants *phyA-211* (Col-0) [58], *phyA-101* (RLD) [59], *phyB-1* (introgressed into RLD) [60,61], *phyA-101 phyB-1* (RLD), *phyA-201* (Ler) [58], *cry1 cry2* (Ler) and *cry1 cry2 phyA-201* (Ler) [62] were described previously. The transgenic lines with constitutively active photoreceptors expressed the phytochromes *A* ^*Y242H*^ and *B* ^*Y276H*^ [7], PHYA406-YFP-DD/NLS [10], CRY1^G380R^ [8] or GUS-CCT1 [9]. The transgenic lines *SPA2*::*SPA1-HA 28*, *SPA2*::*SPA1-HA 70*, *SPA1*::*SPA2-HA 64* and *SPA2*::*SPA2-HA 32* were described previously [42]. The mutants *spa1-7 spa2-1 spa3-1* and *spa1-7 spa3-1 spa4-1* [51] were used whenever no allele information is provided. *spa1-100 spa3-1 spa4-3* [23], *cop1-4 [63]* and *cop1-5* [64] were described. The *spa1-7 spa3-1 spa4-1 phyA-211* quadruple mutant was generated by crossing the *spa1-7 spa3-1 spa4-1* triple mutant with the *phyA-211* single mutant and was confirmed in the F2 and F3 progenies by the *phyA* phenotype and a genotypic analysis using molecular markers that can distinguish between mutant and wild-type *spa* alleles.

To obtain *SPA2*::*SPA2-HA cop1-5 (-/-)* seed, the transgenic line *SPA2*::*SPA2-HA 32* was crossed with *cop1-5 (+/-)*. Transgenic homozygous *cop1-5* seeds were selected in a segregating F4 population based on their black seed phenotype which was scored using a stereo microscope. Seeds with normal seed color served as a control that is homo- or heterozygous for the wild-type *COP1* allele.

LED light sources and seedling growth conditions were as described previously [22,54]. Growth conditions for the *SPA2*::*SPA2-HA cop1-5 (-/-)* experiment were as follows: after stratification of imbibed seeds for 3 days at 4°C, seeds were irradiated with white light for 3 h to break the dormancy and were subsequently kept in darkness for another 21 h. Seeds were then transferred from darkness to Rc (40 μmol m^–2^ s^–1^) for 6 h.

### Generation of transgenic plants expressing ΔCC SPA2-HA

*SPA2*::*ΔCC SPA2-HA* lines express a deletion derivative lacking the amino acids 580–702 in the SPA2 protein. To generate the construct, two PCR fragments were amplified from the full-length *SPA2* ORF lacking the stop codon using the primer pairs SC_SPA2deltaCC_ApaI_F1 and SC_SPA2deltaCC_R1 or SC_SPA2deltaCC_F2 and SPA2deltaN-NotI-R. Both PCR products were purified, combined and subsequently used as templates for amplifying the *ΔCC SPA2* sequence using the primers SC_SPA2deltaCC_ApaI_F1 and SPA2 delta N NotI R, thereby also introducing a 5’ ApaI restriction site and a 3’ NotI restriction site. After PCR-amplification of *ΔCC SPA2*, the resulting fragment was introduced into the pJET1.2 vector (Thermo Scientific). After sequencing of the insert to confirm the correct sequence, the deletion construct was digested with ApaI and NotI and ligated into the ApaI and NotI sites of the pBS vector carrying the *SPA2* 5’ and 3’ regulatory sequences as described previously [42], resulting in the *SPA2*::*ΔCC SPA2* construct in pBS. The 3xHA tag with stop codon was subsequently cloned into the NotI site and the complete insert was cloned into the pJHA212 binary vector [65] as described in [42] to generate *SPA2*::*ΔCC SPA2-HA*. This construct was transformed into *spa1-7 spa2-1 spa3-1* mutant plants by floral dipping. T2 plants were used for analysis.

### Isolation of nuclear protein fractions

In order to detect the native SPA2 protein using an α-SPA2 antibody [42], nuclear proteins were enriched from seedlings as described previously [66].

### Isolation of total proteins

Approximately 200 mg of seedlings or, for *cop1-5* related experiments, approximately 20 μl volume-equivalents of imbibed seeds were homogenized to a fine powder using liquid nitrogen. Lysis buffer [50 mM Tris pH 7.5, 150 mM NaCl, 1 mM EDTA, 10% glycerol, 0.1% Triton X-100, 5 mM DTT, 1% protease inhibitor cocktail (Sigma-Aldrich), 10 μM MG132] was added to the ground tissue at a ratio of 150 μl per 100 mg tissue. The mixtures were thawed on ice and centrifuged at 20.000 g at 4°C for 12 min. 5x Laemmli buffer was added to the supernatant to a final concentration of 1x before heating at 96°C for 5 min. Protein concentrations were determined by Bradford assay (Bio-Rad).

### Immunoblot analysis

For separating nuclear-enriched protein extracts by SDS-PAGE, equal volumes of nuclear-enriched extracts were loaded. To separate total protein extracts, equal amounts of protein were resolved by SDS-PAGE. Protein samples were subsequently blotted onto PVDF membranes. After blotting, membranes were blocked with Rotiblock (Roth) reagent and incubated with the respective primary antibody followed by a horseradish peroxidase (HRP)-conjugated secondary antibody. HRP activity was detected using the SuperSignal West Femto Maximum Sensitivity kit (Thermo Scientific) and visualized by a LAS-4000 Mini bioimager (GE Healthcare Life Sciences). Signal intensities were quantified using Multi-Gauge software (GE Healthcare Life Sciences). Commercial antibodies used were HRP-conjugated α-HA (Roche), α-Histone H3 (Abcam), α-HSC70 (Stressgen), α-α-Tubulin (Sigma-Aldrich), α-rabbit IgG-HRP (Sigma-Aldrich) and α-mouse IgG-HRP (Sigma-Aldrich). α-SPA2 and α-COP1 antibodies were described previously in [42]. α-cry1 [67] and α-cry2 [68] antibodies were used to detect cry1 and cry2, respectively.

### Co-immunoprecipitations

Co-immunoprecipitation experiments were performed using μMACS Anti-HA Starting Kits (Miltenyi Biotec) according to the manufacturer’s protocol with minor modification. Total proteins were extracted as described above. Protein lysates were incubated with 10 μl μMACS Anti-HA MicroBeads. After incubation on ice for 30 min, the mixture was applied onto prepared μ Columns which were placed in the magnetic field of μMACS Separator attached to a MACS MultiStand. The columns were washed four times with lysis buffer and once with Wash Buffer 2 provided by the kit. Elution was performed at 95°C with Elution Buffer according to the manufacturer’s manual. For cry1 and cry2 pull-down experiments, seedlings were pre-infiltrated with 100 μM MG132 and 10 μM clasto-Lactacystin β-lactone twice, 15 min each, before light treatment. Furthermore, five times more protein extract was used for the SPA2-HA immunoprecipitation than for the SPA1-HA immunoprecipitation.

### Hypocotyl length measurement

Seedlings were flattened on the surface of solid MS plates and photographed with a Nikon D5000 digital camera. Images were analyzed by ImageJ 1.43u (Wayne Rasband, National Institutes of Health) to obtain hypocotyl lengths.

### Transcript level analysis

Total RNA isolation, DNase I treatment, first-strand cDNA synthesis and qRT-PCR were performed as described in [42]. Primers used to amplify *HA-tag* and *UBQ10* were previously described [42]. Two biological replicates were included. Relative transcript levels were calculated using the *ΔΔC*_*t*_ method with *UBQ10* as a normalization transcript.

### Accession numbers

COP1 (At2g32950), SPA1 (At2g46340), SPA2 (At4g11110), SPA3 (At3g15354), SPA4 (At1g53090), cry1 (AT4G08920), cry2 (AT1G04400), phyA (AT1G09570), phyB (AT2G18790).

## Supporting Information

S1 Fig SPA2 protein levels in *phyA-201* and in *cry1 cry2 phyA-201* triple mutants. **A.** SPA2 protein levels in 4-day-old dark-grown (D) seedlings of the indicated genotypes that were irradiated with 20 μmol m^–2^ s^–1^ Bc for 0.5 h (B0.5) or 24 h (B24). All mutants are in the Ler accession. **B.** SPA2 protein levels in 4-day-old dark-grown wild-type and *phyA* mutant seedlings that were transferred to FRc (0.1 μmol m^–2^ s^–1^) for 30 min. *phyA-211* is in Col, *phyA-101* in RLD and *phyA-201* in Ler accession. Part of this figure is as in Fig 3A. SPA2 levels were detected in nuclear extracts using an α-SPA2 antibody. Histone H3 levels (H3) served as a loading control.
(PDF)

S2 Fig SPA2 rapidly associates with cry1 in B *in planta*. Co-immunoprecipitation of cry1 by SPA2-HA. 4-day-old dark-grown seedlings expressing SPA2-HA were transferred to 50 μmol m^–2^ s^–1^ B for the indicated time. SPA2-HA proteins were immunoprecipitated using α-HA beads. Seedlings were treated with proteasome inhibitor to prevent SPA2 degradation in Bc. An α-HA antibody was used to detect SPA2-HA protein. An α-cry1 antibody was used to detect cry1. α-Tubulin levels were used as loading control for the input. Asterisks likely indicate phosphorylated cry1.
(PDF)

S3 Fig Expression of the ΔCC SPA2-HA protein does not complement the *spa1 spa2 spa3* mutant phenotype. Visual phenotype of transgenic *spa1-7 spa2-1 spa3-1* seedlings carrying the *SPA2*::*SPA2-HA* or *SPA2*::*ΔCC SPA2-HA* constructs. Seedlings were grown in darkness for 4 days. Numbers refer to independent transgenic lines.
(PDF)

S4 Fig *ΔCC SPA2-HA* transcript levels are not regulated by light. Transcript levels of *SPA2–HA* and *ΔCC SPA2-HA* in transgenic lines grown in darkness or in FRc (5 μmol m^–2^ s^–1^) for 4 days. Expression of *SPA2-HA* and *ΔCC SPA2-HA* was under the control of the *SPA2* promoter. Transcript levels were quantified by qPCR relative to *UBQ10*. Error bars indicate the SEM.
(PDF)

S1 Table Primer list. (DOCX)
