# Supplementary material for: Photoreceptor Specificity in the Light-Induced and COP1-Mediated Rapid Degradation of the Repressor of Photomorphogenesis SPA2 in Arabidopsis
Source: PLoS Genet. 2015 Sep 14;11(9):e1005516. doi: 10.1371/journal.pgen.1005516 (PMC4569408; doi:10.1371/journal.pgen.1005516)
Supplement: S1 Fig — A. SPA2 protein levels in 4-day-old dark-grown (D) seedlings of the indicated genotypes that were irradiated with 20 μmol m–2 s–1 Bc for 0.5 h (B0.5) or 24 h (B24). All mutants are in the Ler accession. B. SPA2 protein levels in 4-day-old dark-grown wild-type and phyA mutant seedlings that were transferred to FRc (0.1 μmol m–2 s–1) for 30 min. phyA-211 is in Col, phyA-101 in RLD and phyA-201 in Ler accession. Part of this figure is as in Fig 3A. SPA2 levels were detected in nuclear extracts using an α-SPA2 antibody. Histone H3 levels (H3) served as a loading control. (PDF) [file pgen.1005516.s001.pdf]

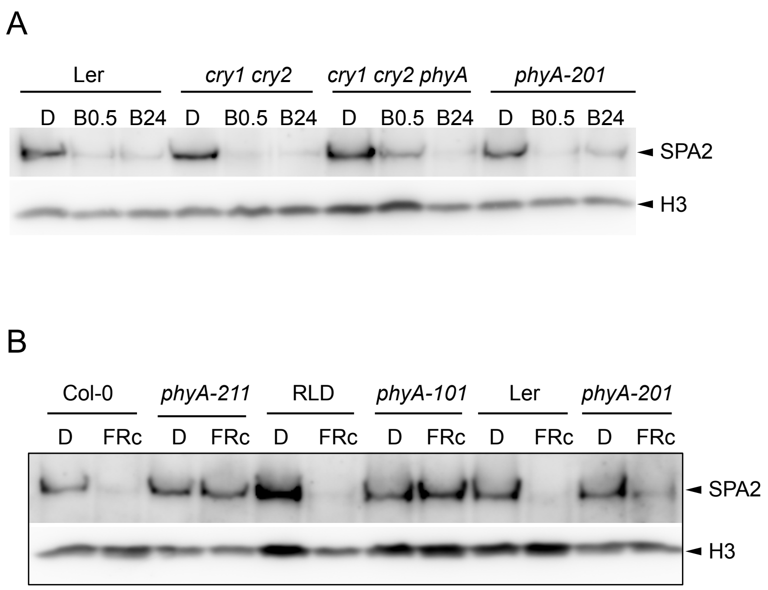

Fig. S1. SPA2 protein levels in *phyA-201* and in *cry1 cry2 phyA-201* triple mutants

A. SPA2 protein levels in 4-day-old dark-grown (D) seedlings of the indicated genotypes that were irradiated with 20 µmol m^{-2} s^{-1} Bc for 0.5 h (B0.5) or 24 h (B24). All mutants are in the Ler accession.

B. SPA2 protein levels in 4-day-old dark-grown wild-type and *phyA* mutant seedlings that were transferred to FRc (0.1 µmol m^{-2} s^{-1}) for 30 min. *phyA-211* is in Col, *phyA-101* in RLD and *phyA-201* in Ler accession. Part of this figure is as in Figure 3A.

SPA2 levels were detected in nuclear extracts using an α-SPA2 antibody. Histone H3 levels (H3) served as a loading control.
