# Supplementary material for: Photoreceptor Specificity in the Light-Induced and COP1-Mediated Rapid Degradation of the Repressor of Photomorphogenesis SPA2 in Arabidopsis
Source: PLoS Genet. 2015 Sep 14;11(9):e1005516. doi: 10.1371/journal.pgen.1005516 (PMC4569408; doi:10.1371/journal.pgen.1005516)
Supplement: S2 Fig — Co-immunoprecipitation of cry1 by SPA2-HA. 4-day-old dark-grown seedlings expressing SPA2-HA were transferred to 50 μmol m–2 s–1 B for the indicated time. SPA2-HA proteins were immunoprecipitated using α-HA beads. Seedlings were treated with proteasome inhibitor to prevent SPA2 degradation in Bc. An α-HA antibody was used to detect SPA2-HA protein. An α-cry1 antibody was used to detect cry1. α-Tubulin levels were used as loading control for the input. Asterisks likely indicate phosphorylated cry1. (PDF) [file pgen.1005516.s002.pdf]

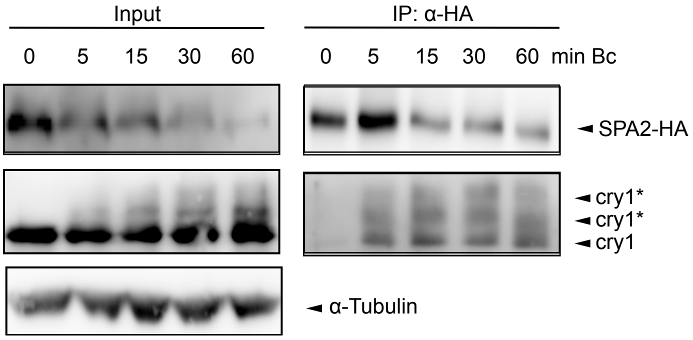

Fig. S2. SPA2 rapidly associates with cry1 in B *in planta*

Co-immunoprecipitation of cry1 by SPA2-HA. 4-day-old dark-grown seedlings expressing SPA2-HA were transferred to 50 µmol m^{-2} s^{-1} B for the indicated time. SPA2-HA proteins were immunoprecipitated using α-HA beads.

Seedlings were treated with proteasome inhibitor to prevent SPA2 degradation in Bc. An α-HA antibody was used to detect SPA2-HA protein. An α-cry1 antibody was used to detect cry1. α-Tubulin levels were used as loading control for the input. Asterisks likely indicate phosphorylated cry1.
