# Supplementary material for: Photoreceptor Specificity in the Light-Induced and COP1-Mediated Rapid Degradation of the Repressor of Photomorphogenesis SPA2 in Arabidopsis
Source: PLoS Genet. 2015 Sep 14;11(9):e1005516. doi: 10.1371/journal.pgen.1005516 (PMC4569408; doi:10.1371/journal.pgen.1005516)
Supplement: S3 Fig — Visual phenotype of transgenic spa1-7 spa2-1 spa3-1 seedlings carrying the SPA2::SPA2-HA or SPA2::ΔCC SPA2-HA constructs. Seedlings were grown in darkness for 4 days. Numbers refer to independent transgenic lines. (PDF) [file pgen.1005516.s003.pdf]

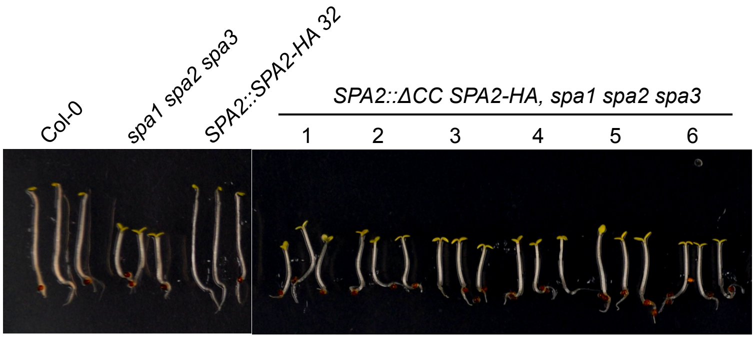

Fig. S3. Expression of the ΔCC SPA2-HA protein does not complement the *spa1 spa2 spa3* mutant phenotype.

Visual phenotype of transgenic *spa1-7 spa2-1 spa3-1* seedlings carrying the *SPA2::SPA2-HA* or *SPA2::ΔCC SPA2-HA* constructs. Seedlings were grown in darkness for 4 days. Numbers refer to independent transgenic lines.
