# Supplementary material for: Photoreceptor Specificity in the Light-Induced and COP1-Mediated Rapid Degradation of the Repressor of Photomorphogenesis SPA2 in Arabidopsis
Source: PLoS Genet. 2015 Sep 14;11(9):e1005516. doi: 10.1371/journal.pgen.1005516 (PMC4569408; doi:10.1371/journal.pgen.1005516)
Supplement: S4 Fig — Transcript levels of SPA2–HA and ΔCC SPA2-HA in transgenic lines grown in darkness or in FRc (5 μmol m–2 s–1) for 4 days. Expression of SPA2-HA and ΔCC SPA2-HA was under the control of the SPA2 promoter. Transcript levels were quantified by qPCR relative to UBQ10. Error bars indicate the SEM. (PDF) [file pgen.1005516.s004.pdf]

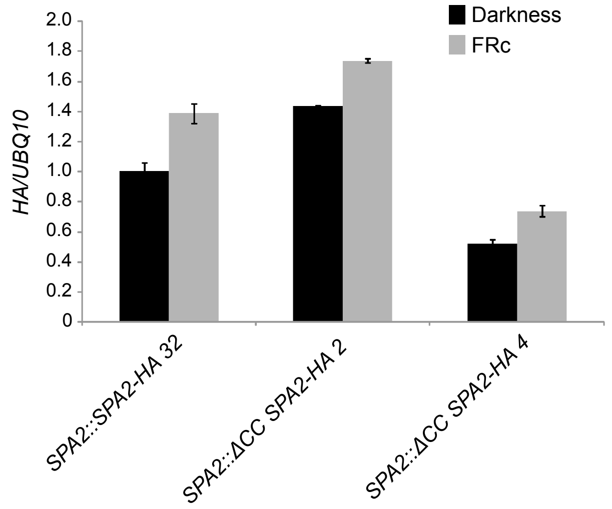


**Fig. S4.** *ΔCC SPA2-HA* transcript levels are not regulated by light.

Transcript levels of *SPA2–HA* and *ΔCC SPA2-HA* in transgenic lines grown in darkness or in FRc (5 µmol $m^{-2}$ $s^{-1}$) for 4 days. Expression of *SPA2-HA* and *ΔCC SPA2-HA* was under the control of the *SPA2* promoter. Transcript levels were quantified by qPCR relative to *UBQ10*. Error bars indicate the SEM.
