# Supplementary material for: Photoreceptor Specificity in the Light-Induced and COP1-Mediated Rapid Degradation of the Repressor of Photomorphogenesis SPA2 in Arabidopsis
Source: PLoS Genet. 2015 Sep 14;11(9):e1005516. doi: 10.1371/journal.pgen.1005516 (PMC4569408; doi:10.1371/journal.pgen.1005516)
Supplement: S1 Table — (DOCX) [file pgen.1005516.s005.docx]

**Table S1:** Primer list

| **Primer name** | **Sequence (5´ to 3´)** |
| --- | --- |
| SPA2 delta N NotI R | TTAAGCGGCCGCGACCAACTGTAGAACTTTGATTG |
| SC_SPA2deltaCC_ApaI_F1 | CCGAGGGCCCATGATGGATGAGGGATCAGT |
| SC_SPA2deltaCC_R1 | CTTACTATATCGAGCAAGGAAATGCTGCAA |
| SC_SPA2deltaCC_F2 | TTGCAGCATTTCCTTGCTCGATATAGTAAG |
| SPA2deltaN-NotI-R | TTAAGCGGCCGCGACCAACTGTAGAACTTTGATTG |
